# Supplementary material for: Crimean-Congo hemorrhagic fever virus antibody prevalence in Mauritanian livestock (cattle, goats, sheep and camels) is stratified by the animal’s age
Source: PLoS Negl Trop Dis. 2021 Apr 12;15(4):e0009228. doi: 10.1371/journal.pntd.0009228 (PMC8081336; doi:10.1371/journal.pntd.0009228)
Supplement: S1 Table — The two Australian samples marked in yellow (Aus 26/31) are the two outliers showing a strongly deviating OD value. (DOCX) [file pntd.0009228.s001.docx]

| **Sample** | **1st run** | | **2nd run** | | **Sample** | **1st run** | | **2nd run** | |
| --- | --- | --- | --- | --- | --- | --- | --- | --- | --- |
|  | **OD value** | **%** | **OD value** | **%** |  | **OD value** | **%** | **OD value** | **%** |
| **Australien camels** | | | | | **Aus 29** | 0,165 | 6% | 0,095 | 3% |
| **Aus 1** | 0,299 | 10% | 0,119 | 4% | **Aus 30** | 0,177 | 6% | 0,079 | 3% |
| **Aus 2** | 0,172 | 6% | 0,053 | 2% | **Aus 31** | 1,561 | 52% | 0,626 | 23% |
| **Aus 3** | 0,105 | 3% | 0,059 | 2% | **Aus 32** | 0,193 | 6% | 0,104 | 4% |
| **Aus 4** | 0,211 | 7% | 0,052 | 2% | **Aus 33** | 0,048 | 2% | 0,025 | 1% |
| **Aus 5** | 0,146 | 5% | -0,001 | 0% | **Aus 34** | 0,035 | 1% | 0,037 | 1% |
| **Aus 6** | 0,141 | 5% | 0,063 | 2% | **Aus 35** | 0,072 | 2% | 0,016 | 1% |
| **Aus 7** | 0,135 | 4% | 0,034 | 1% | **Aus 36** | 0,212 | 7% | 0,077 | 3% |
| **Aus 8** | 0,201 | 7% | 0,066 | 2% | **Aus 37** | 0,035 | 1% | -0,012 | 0% |
| **Aus 9** | 0,098 | 3% | 0,076 | 3% | **Aus 40** | 0,071 | 2% | 0,018 | 1% |
| **Aus 10** | 0,235 | 8% | 0,134 | 5% | **Aus 41** | 0,034 | 1% | 0,015 | 1% |
| **Aus 11** | 0,084 | 3% | 0,057 | 2% | **Aus 42** | 0,023 | 1% | -0,011 | 0% |
| **Aus 12** | 0,072 | 2% | 0,035 | 1% | **Aus 43** | 0,037 | 1% | -0,005 | 0% |
| **Aus 13** | 0,063 | 2% | -0,010 | 0% | **Aus 44** | 0,037 | 1% | 0,027 | 1% |
| **Aus 14** | 0,070 | 2% | 0,030 | 1% | **Aus 45** | 0,083 | 3% | -0,002 | 0% |
| **Aus 15** | 0,037 | 1% | 0,048 | 2% | **Aus 46** | 0,156 | 5% | 0,029 | 1% |
| **Aus 16** | -0,028 | -1% | 0,014 | 1% | **German camels from zoos** | | | | |
| **Aus 17** | 0,073 | 2% | 0,035 | 1% | **Ger 1** | 0,064 | 3% |  |  |
| **Aus 18** | 0,054 | 2% | 0,023 | 1% | **Ger 2** | 0,108 | 5% |  |  |
| **Aus 19** | 0,068 | 2% | -0,007 | 0% | **Ger 3** | 0,045 | 2% |  |  |
| **Aus 20** | 0,040 | 1% | -0,026 | -1% | **Ger 4** | 0,037 | 2% |  |  |
| **Aus 21** | -0,002 | 0% | -0,001 | 0% | **Ger 5** | 0,066 | 3% |  |  |
| **Aus 22** | 0,136 | 4% | 0,094 | 4% | **Ger 6** | 0,153 | 7% |  |  |
| **Aus 23** | 0,151 | 5% | 0,066 | 2% | **Ger 7** | 0,015 | 1% |  |  |
| **Aus 24** | 0,203 | 7% | 0,112 | 4% | **Ger 8** | 0,037 | 2% |  |  |
| **Aus 25** | 0,181 | 6% | 0,104 | 4% | **Ger 9** | 0,035 | 2% |  |  |
| **Aus 26** | 2,443 | 82% | 1,172 | 43% | **Ger 10** | 0,084 | 4% |  |  |
| **Aus 27** | 0,187 | 6% | 0,111 | 4% | **Ger 11** | -0,009 | 0% |  |  |
| **Aus 28** | 0,135 | 5% | 0,089 | 3% | **Ger 12** | 0,051 | 2% |  |  |
