# Supplementary material for: Crimean-Congo hemorrhagic fever virus antibody prevalence in Mauritanian livestock (cattle, goats, sheep and camels) is stratified by the animal’s age
Source: PLoS Negl Trop Dis. 2021 Apr 12;15(4):e0009228. doi: 10.1371/journal.pntd.0009228 (PMC8081336; doi:10.1371/journal.pntd.0009228)
Supplement: Attachment — Submitted filename: CCHFV Serology_Schulz_Rebuttal letter2.doc [file pntd.0009228.s002.doc]

| To  Editorial Office of “Plos Neglected Tropical Diseases” Journal  Editor-in-Chief |  | **Institute of Novel and Emerging Infectious Diseases**  Prof. Dr. Martin H. Groschup | | | |  |
| --- | --- | --- | --- | --- | --- | --- |
|  |  | | | |  | |
|  | Telefone: | | | 038351-7-1163 | | |
|  | Fax: | | | 038351-7-1191 | | |
|  | E-Mail: | | | martin.groschup@fli.de | | |
|  | Date: | | 25. January 2021 | | | |

Dear Editors,

Please find enclosed the revised manuscript entitled *“*Crimean-Congo hemorrhagic fever virus antibody prevalence in Mauritanian livestock (cattle, goats, sheep and camels) is stratified by the animal’s age”*.* The manuscript was modified according to the valuable suggestions of the reviewers and editor. We greatly appreciate the opportunity that our manuscript will be subject to review again.

Sincerely yours,

Prof. Dr. Martin H. Groschup (for all co-authors)

**Reviewer's Responses to Questions**

**General remarks:**

Thank you for submitting your paper, "Crimean-Congo hemorrhagic fever virus antibody prevalence in Mauritanian livestock (cattle, goats, sheep and camels) is stratified by the animal’s age", to PLOS Neglected Tropical Diseases. We have checked over your submission and, before we can assign your manuscript to the Editor, we ask that you first please make the following modifications:

1. We note that the following authors have not yet confirmed their authorship on this manuscript: Yahya Barry, Aliou Ba, Mohamed L. Haki, Miriam A. Sas, Baba A. Doumbia, Mohamed Y. Bah

At this point this request is not urgent; we have re-sent their co-author confirmation email containing an auto-confirmation link for them to click, and we will reach out to them at a later stage if we don't hear from them. We wanted to mention this in case you are aware of them experiencing technical issues with confirming authorship via the link. If so, please do ask them to contact us directly via email and we'd be happy to assist.

Authors reply:

Once again, we have asked all the above-mentioned co-authors to confirm the email.

2. We note that several of your files are duplicated on your submission. Please remove any unnecessary or old files from your revision, and make sure that only those relevant to the current version of the manuscript are included.

Authors reply:

We removed all duplicated files and texts on our submission.

3. We do not publish any copyright or trademark symbols that usually accompany proprietary names, eg (R), (C), or TM (e.g. next to drug or reagent names). Therefore please remove all instances of trademark/copyright symbols throughout the text, including "Infinite®" on page 7.

Authors reply:

All trademark symbols in the text were removed.

4. Thank you for including your ethics statement: [The sample collection was carried out by the Mauritanian State Veterinary Laboratory ONARDEL (Office National de Recherche et de Développement de l'Elevage) following following all relevan national as well as international regulations and according to fundamental ethical principles for diagnostic purposes in the framework of a governmental program for the animal health surveillance.]. Please include the approval number(s), or a statement that approval was granted by the named board(s)

Authors reply:

The samples were taken by ONARDEL in order to fulfill its governmental mandate to conduct livestock animal monitoring and surveillance programs for veterinary and zoonotic pathogens.

5. Your manuscript is missing the following sections: [Introduction]. Please ensure these are present, and in the correct order, and that any references to subheadings in your main text are correct. An outline of the required sections can be consulted in our submission guidelines here: [https://scanmail.trustwave.com/?c=4477&d=56qR4A4aZOR1g7hTHguEGbUIqHvUWLayjRqLk9-Qkw&u=https%3a%2f%2fjournals%2eplos%2eorg%2fplosntds%2fs%2fsubmission-guidelines%23loc-parts-of-a-submission](https://scanmail.trustwave.com/?c=4477&d=56qR4A4aZOR1g7hTHguEGbUIqHvUWLayjRqLk9-Qkw&u=https%3A%2F%2Fjournals.plos.org%2Fplosntds%2Fs%2Fsubmission-guidelines%23loc-parts-of-a-submission)

Authors reply:

The section “Background” was renamed into “Introduction” in the manuscript.

6. Please provide separate figure files in .tif or .eps format only and remove any figures embedded in your manuscript file. Please ensure that all files are under our size limit of 20MB.

For more information about how to convert your figure files please see our guidelines: Once you've converted your files to .tif or .eps, please also make sure that your figures meet our format requirements

[https://scanmail.trustwave.com/?c=4477&d=56qR4A4aZOR1g7hTHguEGbUIqHvUWLayjUCPlNzGwg&u=https%3a%2f%2fjournals%2eplos%2eorg%2fplosntds%2fs%2ffigures](https://scanmail.trustwave.com/?c=4477&d=56qR4A4aZOR1g7hTHguEGbUIqHvUWLayjUCPlNzGwg&u=https%3A%2F%2Fjournals.plos.org%2Fplosntds%2Fs%2Ffigures)

[https://scanmail.trustwave.com/?c=4477&d=56qR4A4aZOR1g7hTHguEGbUIqHvUWLayjRaIk4iXwg&u=https%3a%2f%2fjournals%2eplos%2eorg%2fplosntds%2fs%2ffigures%23loc-file-requirements](https://scanmail.trustwave.com/?c=4477&d=56qR4A4aZOR1g7hTHguEGbUIqHvUWLayjRaIk4iXwg&u=https%3A%2F%2Fjournals.plos.org%2Fplosntds%2Fs%2Ffigures%23loc-file-requirements)

Authors reply:

Figures 1 and 2 have been removed from the manuscript and provided as separate figure files.

7. We have noticed that you have uploaded supporting information but you have not included a list of legends. Please add a full list of legends for all supporting information files (including figures, table and data files) after the references list.

Authors reply:

We added a figure legend for the supporting information after the reference list.

8. When completing the data availability statement of the submission form, you indicated that "All relevant data are within the manuscript and its Supporting Information files.". However, you also checked the following box: “Tick here if the URLs/accession numbers/DOIs will be available only after acceptance of the manuscript for publication so that we can ensure their inclusion before publication.” If this box was checked in error, and there is no additional data to deposit, please ensure this box is unticked when you resubmit.

Authors reply:

Thanks a lot for this advice. The tick in the box has been removed.

9. Thank you for your Financial Disclosure statement:

[This study was co-funded by the German Office for Foreign Affairs (German Partnership Program for Biosecurity), by the Deutsche Forschungsgemeinschaft (grant number: GR 980/4-1) as well as by the European Union (LEAP-AGRI LEARN project (ID 222). The funders had no role in study design, data collection and analysis, decision to publish, or preparation of the manuscript.

[https://scanmail.trustwave.com/?c=4477&d=56qR4A4aZOR1g7hTHguEGbUIqHvUWLayjRTZwtvMmQ&u=https%3a%2f%2fwww%2edfg%2ede%2f](https://scanmail.trustwave.com/?c=4477&d=56qR4A4aZOR1g7hTHguEGbUIqHvUWLayjRTZwtvMmQ&u=https%3A%2F%2Fwww.dfg.de%2F)

[https://scanmail.trustwave.com/?c=4477&d=56qR4A4aZOR1g7hTHguEGbUIqHvUWLayjRqLkovFww&u=https%3a%2f%2fwww%2eauswaertiges-amt%2ede%2fde%2f%5d](https://scanmail.trustwave.com/?c=4477&d=56qR4A4aZOR1g7hTHguEGbUIqHvUWLayjRqLkovFww&u=https%3A%2F%2Fwww.auswaertiges-amt.de%2Fde%2F%5D)

Please include the initials, alongside each funding source, of each author to receive each grant.

Authors reply:

The initials of the authors were added alongside the funding sources.

Please note that all figures and supporting information files will be published under the Creative Commons Attribution License ([http://scanmail.trustwave.com/?c=4477&d=zPuQ4GG_WtdvYgC_jf0yvHzMQ1vnBR4bcF3WHc5y2Q&u=http%3a%2f%2fcreativecommons%2eorg%2flicenses%2fby%2f4%2e0%2f%29](http://scanmail.trustwave.com/?c=4477&d=zPuQ4GG_WtdvYgC_jf0yvHzMQ1vnBR4bcF3WHc5y2Q&u=http%3A%2F%2Fcreativecommons.org%2Flicenses%2Fby%2F4.0%2F)) Under this license, authors retain ownership of the copyright for their article, but allow anyone to download, reuse, reprint, modify, distribute, and/or copy articles in PLOS journals for any reason, even commercially, so long as the original authors and source are cited. Permission is not required from PLOS or authors to reuse any content as long as proper attribution is provided.

We ask that you please confirm that you either created the following or have written permission from the copyright holder to publish them under a CC-BY license:

Figure 1

We have noticed that your manuscript contains a map as one of your figures. Please ensure that this map was published under a CC-BY license ([http://scanmail.trustwave.com/?c=4477&d=zPuQ4GG_WtdvYgC_jf0yvHzMQ1vnBR4bcFWAHJsnhw&u=http%3a%2f%2fjournals%2eplos%2eorg%2fploscompbiol%2fs%2fcontent-license](http://scanmail.trustwave.com/?c=4477&d=zPuQ4GG_WtdvYgC_jf0yvHzMQ1vnBR4bcFWAHJsnhw&u=http%3A%2F%2Fjournals.plos.org%2Fploscompbiol%2Fs%2Fcontent-license)) - maps licensed under any other license will NOT be compatible with our full re-use CC-BY license.

The following websites are good examples of where you can source open access or public domain maps:

* World of Maps - Many, but not all, of the maps are in the public domain so please check the usage rights before downloading. ([http://scanmail.trustwave.com/?c=4477&d=zPuQ4GG_WtdvYgC_jf0yvHzMQ1vnBR4bcF2ETpxxjw&u=http%3a%2f%2fwww%2eworldofmaps%2enet](http://scanmail.trustwave.com/?c=4477&d=zPuQ4GG_WtdvYgC_jf0yvHzMQ1vnBR4bcF2ETpxxjw&u=http%3A%2F%2Fwww.worldofmaps.net))

* U.S. Geological Survey (USGS) - All maps are in the public domain. ([http://scanmail.trustwave.com/?c=4477&d=zPuQ4GG_WtdvYgC_jf0yvHzMQ1vnBR4bcF7WGMp13A&u=http%3a%2f%2fwww%2eusgs%2egov](http://scanmail.trustwave.com/?c=4477&d=zPuQ4GG_WtdvYgC_jf0yvHzMQ1vnBR4bcF7WGMp13A&u=http%3A%2F%2Fwww.usgs.gov))

([http://scanmail.trustwave.com/?c=4477&d=zPuQ4GG_WtdvYgC_jf0yvHzMQ1vnBR4bcF2ETs93jw&u=http%3a%2f%2fglovis%2eusgs%2egov%2f%29](http://scanmail.trustwave.com/?c=4477&d=zPuQ4GG_WtdvYgC_jf0yvHzMQ1vnBR4bcF2ETs93jw&u=http%3A%2F%2Fglovis.usgs.gov%2F))

([http://scanmail.trustwave.com/?c=4477&d=zPuQ4GG_WtdvYgC_jf0yvHzMQ1vnBR4bcAzWScN93g&u=http%3a%2f%2flandsatlook%2eusgs%2egov%2f](http://scanmail.trustwave.com/?c=4477&d=zPuQ4GG_WtdvYgC_jf0yvHzMQ1vnBR4bcAzWScN93g&u=http%3A%2F%2Flandsatlook.usgs.gov%2F))

* PlaniGlobe - All maps are published under a Creative Commons license so please cite “PlaniGlobe, [http://scanmail.trustwave.com/?c=4477&d=zPuQ4GG_WtdvYgC_jf0yvHzMQ1vnBR4bcF_WTMt1iw&u=http%3a%2f%2fwww%2eplaniglobe%2ecom](http://scanmail.trustwave.com/?c=4477&d=zPuQ4GG_WtdvYgC_jf0yvHzMQ1vnBR4bcF_WTMt1iw&u=http%3A%2F%2Fwww.planiglobe.com) CC BY 2.0” in the image credit after the caption. ([http://scanmail.trustwave.com/?c=4477&d=zPuQ4GG_WtdvYgC_jf0yvHzMQ1vnBR4bcF6HTc8m2Q&u=http%3a%2f%2fwww%2eplaniglobe%2ecom%2f%3flang%3denl](http://scanmail.trustwave.com/?c=4477&d=zPuQ4GG_WtdvYgC_jf0yvHzMQ1vnBR4bcF6HTc8m2Q&u=http%3A%2F%2Fwww.planiglobe.com%2F%3Flang%3Denl))

* Natural Earth - All maps are public domain. ([http://scanmail.trustwave.com/?c=4477&d=zPuQ4GG_WtdvYgC_jf0yvHzMQ1vnBR4bcFvUHZ583A&u=http%3a%2f%2fwww%2enaturalearthdata%2ecom%2fabout%2fterms-of-use%2f](http://scanmail.trustwave.com/?c=4477&d=zPuQ4GG_WtdvYgC_jf0yvHzMQ1vnBR4bcFvUHZ583A&u=http%3A%2F%2Fwww.naturalearthdata.com%2Fabout%2Fterms-of-use%2F))

* NASA - All maps are public domain. ([https://scanmail.trustwave.com/?c=4477&d=zPuQ4GG_WtdvYgC_jf0yvHzMQ1vnBR4bcFqDGJgj2w&u=https%3a%2f%2feol%2ejsc%2enasa%2egov%2f%29](https://scanmail.trustwave.com/?c=4477&d=zPuQ4GG_WtdvYgC_jf0yvHzMQ1vnBR4bcFqDGJgj2w&u=https%3A%2F%2Feol.jsc.nasa.gov%2F))

([http://scanmail.trustwave.com/?c=4477&d=zPuQ4GG_WtdvYgC_jf0yvHzMQ1vnBR4bcF3WHpx0jg&u=http%3a%2f%2fearthobservatory%2enasa%2egov%2f](http://scanmail.trustwave.com/?c=4477&d=zPuQ4GG_WtdvYgC_jf0yvHzMQ1vnBR4bcF3WHpx0jg&u=http%3A%2F%2Fearthobservatory.nasa.gov%2F)) ([http://scanmail.trustwave.com/?c=4477&d=zPuQ4GG_WtdvYgC_jf0yvHzMQ1vnBR4bcA7TTM5xjg&u=http%3a%2f%2flandsat%2egsfc%2enasa%2egov%2fimages%2f](http://scanmail.trustwave.com/?c=4477&d=zPuQ4GG_WtdvYgC_jf0yvHzMQ1vnBR4bcA7TTM5xjg&u=http%3A%2F%2Flandsat.gsfc.nasa.gov%2Fimages%2F))

* Wikimedia Commons - Many, but not all, of the maps are licensed under compatible licenses so please check the usage rights before downloading. ([https://scanmail.trustwave.com/?c=4477&d=zPuQ4GG_WtdvYgC_jf0yvHzMQ1vnBR4bcFXXH8sm2w&u=https%3a%2f%2fcommons%2ewikimedia%2eorg%2fwiki%2fAtlas%5fof%5fthe%5fworld](https://scanmail.trustwave.com/?c=4477&d=zPuQ4GG_WtdvYgC_jf0yvHzMQ1vnBR4bcFXXH8sm2w&u=https%3A%2F%2Fcommons.wikimedia.org%2Fwiki%2FAtlas_of_the_world))

Please provide written confirmation via return email. It must clarify that the copyright holder understands and agrees to the terms of this license.

Once you have made the necessary changes in Editorial Manager, please re-approve the submission by following the same steps as before.

If you have any questions or require assistance, please don't hesitate to get in touch. To help us minimize inbox volumes, we kindly ask that you not reply to this email simply to confirm you have resubmitted your revisions.

Authors reply:

We forgot to add which programme was used to create the map. The following sentence was added in the figure legend of Fig. 1.

*“The map was created with ArcGIS 10.5.1 (ESRI, Redlands, CA, USA).”*

<b>Key Review Criteria Required for Acceptance?</b></br></br>

As you describe the new analyses required for acceptance, please consider the following:</br></br>

<b>Methods</b></br></br>

-Are the objectives of the study clearly articulated with a clear testable hypothesis stated?</br>

-Is the study design appropriate to address the stated objectives?</br>

-Is the population clearly described and appropriate for the hypothesis being tested?</br>

-Is the sample size sufficient to ensure adequate power to address the hypothesis being tested?</br>

-Were correct statistical analysis used to support conclusions?</br>

-Are there concerns about ethical or regulatory requirements being met?</br></br>

**Reviewer #1:** Camel ELISA is well validated. Positive samples were confirmed in repeat measurements. Samples collected from across the country.

Authors reply:

Thanks a lot for your positive feedback, which we appreciate.

**Reviewer #2:** Please provide more detailed information regarding the sampling framework in your Methods section:

What considerations were made in determining the sample sizes? In each geographic region, how were individual animals chosen for sampling? Were they all sampled from a single herd/farm? Why were some animals only sampled from certain regions and not others?

Authors reply:

A completely new paragraph was added as part of the revision of the chapter "Sampling sites and serum collection" (LL. 107-116). We have tried to address all your questions/comments in this section.

*“Within the regions, one to a maximum of three different flocks from local farmers were sampled. Considering the relatively small size of the sampled flocks, we decided to consolidate the animals region-wise. The sampling region "Nouakchott" constitutes an exception, since the samples were taken in the most important slaughterhouse / livestock market. Livestock from all over the country were driven up to this market/slaughter house. Given the large size of the country and the lack of infrastructure especially in rural the areas, it was not feasible to take samples from all species in each region. Besides, political conflicts in the border regions and the partly limited compliance of livestock owners also impaired the sampling framework. Therefore, a ''convenience sampling'' was performed.“*

Why was CCHFV strain Hoti utilized as the antigenic component of the Camel ELISA? Why not an African strain like IbAr10200 or UG3010? It would be nice to see a comment on this because some data suggest that even NP-based ELISA's for CCHF may lose sensitivity based on antigenic (geographic) variation (PMID: 23171700).

Authors reply:

Thank very much you for your reasonable question. We have added both a sentence in the material/methods section (LL.125-127) as well as included a completely new paragraph into the discussion (LL.296-302). We hope that it is now more understandable why we used a "European" antigen.

*“This same protein was used as the antigenic component in the other in-house assays for cattle, sheep and goats described in the section below.”*

and

*“In general, serological testing for CCHFV antibodies can be considered as a challenging issue. As there are many different factors influencing the assay, findings should be interpreted carefully. Besides cross-reactivity with other Orthonairoviruses, one of the major challenges is posed by the large genetic variability linked to the geographical distribution of the virus. Currently, there is no serological assay available, which can cover this broad range of genetic diversity in terms of antigenic components used in one single test. Therefore, it has to be emphasized that our in-house assays utilized antigens from Eurasian CCHFV strains.”*

--------------------

<b>Results</b></br></br>

-Does the analysis presented match the analysis plan?</br>

-Are the results clearly and completely presented?</br>

-Are the figures (Tables, Images) of sufficient quality for clarity?</br></br>

**Reviewer #1:** Results are clearly presented.

Authors could consider presenting their seroprevalence data on the map of figure 1 to give a better spatial understanding of the data.

**Reviewer #2:** Please provide more detailed information/legend for Figure 1 (see Methods section comments).

Authors reply to Reviewer 1 and 2

Following careful consideration, we decided not to include all prevalences into the map, as it would otherwise become too convoluted. However, we have changed the map (Fig. 1) in a way that it is now visible how many animals have been sampled in each region and what proportion the different species made up. We have also adapted the legend and figure caption.

--------------------

<b>Conclusions</b></br></br>

-Are the conclusions supported by the data presented?</br>

-Are the limitations of analysis clearly described?</br>

-Do the authors discuss how these data can be helpful to advance our understanding of the topic under study?</br>

-Is public health relevance addressed?</br></br>

**Reviewer #1:** Conclusions are well supported, authors discuss data in context of other data thoroughly and provide discussion on potential explanations for their findings.

Authors reply:

Thank you for your positive feedback.

**Reviewer #2:** The Discussion section references previous studies that have shown nomadic grazing can significantly impact CCHF seroprevalences (Lines 312-313). Can you comment on how this relates to your dataset (or not)? Please describe the type of livestock farming practices that are used in Mauritania (nomadic pastoralism? stationary, irrigated agricultural practices? mix of both?). Do these practices vary by the regions you sampled?

Lines 314-316: You mention vegetation or lack of tick treatments as possible parameters that could account for higher CCHFV prevalence in cattle/camels vs sheep/goats. Do you have any additional information to help lend support to these hypotheses? Is it possible comment on differences in vegetation/agroecological zones (AEZs) between sample sites or regions? What about differences in acaricide practices?

Authors reply:

We added a new paragraph in the material/method section (LL. 97-103) describing the typical livestock practices in Mauritania. Furthermore, we have added a section in the discussion (LL. 358-363) that describes the potential impact of husbandry practices and the use of acaricides on seroprevalence. Unfortunately, we don’t have detailed information on the treatment of the animals examined and can thus only provide a general statement.

*„It is dominated by a very dry, hot and windy climate. Notable amounts of rainfall in terms of a rainy season occur only in the most southern border regions of the country, which extend to the Sahel.* *Livestock farming is mainly practiced in the form of nomadic pastoralism and semi-extensive husbandry. Solely stationary, irrigated agricultural practices /stable housing systems do not play a major role and are only rarely practiced. For this reason, breeding of cattle less adapted to drought and hot climate is mainly found in the south of the country, while small ruminants and camels are also kept in the northern regions [17, 18].”*

and

*„In general, the husbandry of camels in Mauritania differs from the small ruminants. Camels are extensively kept and spend almost their entire live grazing in the bushlands. In contrast, sheep and goats are bred semi-extensively and spend less time on the pasture. They also receive more frequently veterinary treatment (antiparasitics etc.) than camels. Although there is no detailed information on the treatment of the sampled animals available, these parameters may also account for higher CCHFV prevalences in cattle and camels compared to sheep and goats of same age groups as well as for the deviating serological findings among the different regions.”*

Some of the regional differences in CCHFV seroprevalence are interesting, for example, cattle in Hodh El Chargui. It would be useful to point this out & have a short discussion about why this might be.

Authors reply:

During a thorough check of the age information of the animals, we noticed that the overall average age of cattle was calculated incorrectly. It is actually 5.84 years instead of 10.17 years (Table 2). However, since this was a solitary calculation error, it had no impact on the statistical analyses conducted! Based on this new value (5.84 years), the average age of the cattle in Hodh El Chargui (7.24 years) is considerably higher than the overall average age and thus consistent with the general statements of the manuscript. It has been included in the discussion (LL. 324-326).

*“Interestingly, the average age of tested cattle in Hodh el Chargui (7.24 years), which had a high prevalence of 95%, was also considerably higher than the overall average age of all examined bovines (5.84 years; Tab. 2 and 3).”*

--------------------

<b>Editorial and Data Presentation Modifications?</b></br><br/>

**Reviewer #2:** Lines 70-71: Please expand on this & briefly describe the type of livestock farming practices that are used in Mauritania. Nomadic pastoralism? More stationary, irrigated agricultural practices? or a mix of both?

Authors reply:

As mentioned above, the material/methods section has been substantially revised accordingly and a description of livestock practices in Mauritania has been added.

Line 74 mentions a "first" CCHFV serological study on cattle in Muaritania in 1983. Then, Lines 77-78 mention that cattle were tested for IgG antibodies for the first time in 2013. This is a bit confusing. What were they measuring in the 1983 study? Just a quick specification in the text would help.

Authors reply:

Thanks for the advice. You are right it, was written in a slightly confusing way. The text has been adapted accordingly (LL. 80-81).

*“In 2013, in a first comprehensive study, cattle were tested solely for CCHFV IgG antibodies and a surprisingly high seropravalence of 67 % was observed [12]. Investigations of cattle in Sudan [13] showed that collection site, age, husbandry system and tick infestation are the biggest risk factors for CCHFV seroprevalence.”*

**Reviewer #3:** Data from negative control sera from Australia and German are not presented. These data should be included in supplement or included in the main figures or tables. Also, how many samples were considered "inconclusive" and please include the results from IFA data as well.

Authors reply:

We have added a new supplementary table (Sup. Table. 1), which provides an overview of the tested Australian and German camels. The IFA results have been added to the results section (LL. 218-219). Please note that the inconclusive results of the camels were already included in Table 1.

*“In total, thirty-two inconclusive sera from cattle (8/13), goats (1/13), and sheep (0/6) were additionally tested in the IFA, of which nine were finally considered positive. “*

--------------------

<b>Summary and General Comments</b></br></br>

Use this section to provide overall comments, discuss strengths/weaknesses of the study, novelty, significance, general execution and scholarship. You may also include additional comments for the author, including concerns about dual publication, research ethics, or publication ethics. If requesting major revision, please articulate the new experiments that are needed.

**Reviewer #1:** The manuscript by Schulz et al is well written and data is clearly presented. Author's data adds to the body of knowledge on the wide prevalence of CCHFV throughout Africa but add insight onto how animal age impacts seroprevalence. Authors provide key validation data on their in-house ELISA and their conclusions are well supported. Authors discuss their results in the context of previous findings thoroughly.

Minor Comments:

Authors found that camels even at a young age were significantly more likely to be sero-positive than goats or sheep. Authors discuss that this could be due to husbandry practices. Is it known if the local population indeed have different husbandry practices for these livestock species? In regions with multiple species, is it typical for a farmer or family to have multiple species?

Authors reply:

As described above, we have added a paragraph on livestock husbandry practices in Mauritania. It is indeed common that sometimes more than one species is kept simultaneously by the same farmer. Unfortunately, we do not have detailed information on the sampled animals, and thus cannot make a definite statement in this respect.

Some editing for grammar and syntax is needed. Ex. Lines 294-295, 290-292

Authors reply:

The sentences were rephrased in the mentioned lines (now LL. 332-335 and LL. 337-338). We also changed the wording in Line 59-61.

*“Obviously, higher age coincides with a greater chance of exposure to CCHFV-positive ticks in endemic regions and thus becoming infected with the virus.”*

and

*“Animal infection trials in sheep demonstrated IgG antibody titers that persisted up to 30 respectively 40 days post- infection [33, 34].”*

and

*“Furthermore, findings of this study contribute to a better understanding of the current epidemiological CCHFV situation in sub-Saharan Africa and which role different livestock species play regarding the viral circulation in endemic regions. “*

Under what context were the samples outside of the capital region collected? Markets, farms, slaughterhouses?

How does the climate of the country differ across the regions?

Authors reply:

The sample collection was described more detailed in lines 108-116 and the climate of Mauritania in lines 97-100. Since we did not have a comparable number of samples from each species across the different climate zones, it is difficult to draw meaningful conclusions about the climate influence on our seroprevalence.

*“Within the regions, one to a maximum of three different flocks from local farmers were sampled. Considering the relatively small size of the sampled flocks, we decided to consolidate the animals region-wise. The sampling region "Nouakchott" constitutes an exception, since the samples were taken in the most important slaughterhouse / livestock market. Livestock from all over the country were driven up to this market/slaughter house. Given the large size of the country and the lack of infrastructure especially in rural the areas, it was not feasible to take samples from all species in each region. Besides, political conflicts in the border regions and the partly limited compliance of livestock owners also impaired the sampling framework. Therefore, a ''convenience sampling'' was performed. “*

and

*“It is dominated by a very dry, hot and windy climate. Notable amounts of rainfall in terms of a rainy season occur only in the most southern border regions of the country, which extend to the Sahel. Livestock farming is mainly practiced in the form of nomadic pastoralism and semi-extensive husbandry.”*

**Reviewer #3:** This manuscript describe the development of an ELISA capable of detecting CCHFV antibody in camel. Rigorous validation was performed to ensure the robustness of the assay. A serosurvey was performed in sheep, cattle, camels and goats in several regions of Mauritania. The data analysis revealed that CCHFV seropositivity is strongly linked to age rather than species. I only have minor edits that can be addressed in a revised manuscript.

1) Abstract :"The reservoir and vector are ticks of the genus Hyalomma. Livestock animals (such as cattle, small ruminants and camels) develop a viremias..", replace "reservoir" by "main reservoir".

Authors reply:

*The wording was changed accordingly.*

2) "he upper cut-off value ( = 1, = 2) was set to 19.96 % and samples above this value were considered to be CCHFV positive. The lower cut-off value ( = 1, = 2) was 10.45 % and samples below this value were considered as negative"

There seem to be a mistake in f value here?

Authors reply:

*Thank you for pointing out this mistake. The value for "f" for the lower cut-off was corrected from "2" to "1".*
